# Computed tomography in children with community-acquired pneumonia

**DOI:** 10.1007/s00247-017-3891-0

**Published:** 2017-09-21

**Authors:** Savvas Andronikou, Pierre Goussard, Erich Sorantin

**Affiliations:** 1 Department of Paediatric Radiology, Bristol Royal Hospital for Children and the University of Bristol, Upper Maudlin Street, Bristol, BS2 8BJ UK; 2 Department of Radiology, University of Cape Town, Cape Town, South Africa; 3 Department of Paediatrics and Child Health, Tygerberg Hospital, Stellenbosch University, Cape Town, South Africa; 4 Department of Radiology, Medical University Graz, Graz, Austria

**Keywords:** Children, Computed tomography, Empyema, Lung, Lung abscess, Necrotizing pneumonia, Pneumonia

## Abstract

Diagnostic imaging plays a significant role in both the diagnosis and treatment of complications of pneumonia in children and chest radiography is the imaging modality of choice. Computed tomography (CT) on the other hand, is not currently a first-line imaging tool for children with suspected uncomplicated community-acquired pneumonia and is largely reserved for when complications of pneumonia are suspected or there is difficulty in differentiating pneumonia from other pathology. This review outlines the situations where CT needs to be considered in children with pneumonia, describes the imaging features of the parenchymal and pleural complications of pneumonia, discusses how CT may have a wider role in developing countries where human immunodeficiency virus (HIV) and tuberculosis are prevalent, makes note of the role of CT scanning for identifying missed foreign body aspiration and, lastly, addresses radiation concerns.

## Introduction

There has been a significant increase in hospital admissions for complicated community-acquired pneumonia both in the developed world and the developing world [1]. Contributing to this is the high incidence of human immunodeficiency virus (HIV) infection and tuberculosis in the developing world, resulting in overlapping presentations, treatment failure and difficulty making clinical diagnoses. Children with pneumonia who do not respond appropriately to treatment should be investigated for any suppurative parenchymal complications that may have developed [2]. According to the British Thoracic Society Guidelines [3], if a child remains feverish or unwell 48 h after the start of treatment, the child must be re-evaluated regarding a possible complication. There is a spectrum of suppurative parenchymal complications of pneumonia [4]. The Pediatric Infectious Diseases Society and the Infectious Diseases Society of America list the following complications associated with community-acquired pneumonia in children [5]: pleural effusion or empyema, pneumothorax, lung abscess, bronchopleural fistula and necrotizing pneumonia. Such complications are reported in up to 53% of children hospitalized with pneumonia [6]. Harris et al. [6] indicate that children are predisposed to more severe forms of lung infection resulting in suppuration and lung abscess. Among predisposing factors are underlying congenital abnormalities such as cysts and sequestrations, bronchiectasis, neurological disorders and immune deficiencies [3]. Also noted are organisms responsible for causing necrotizing pneumonia and lung abscess more frequently -- these include serotypes of pneumococcus and *S. aureus* [3]. This is due, in part, to a “spectral shift of pneumococcal strains after the introduction of the pneumococcal vaccine and the emergence of methicillin-resistance *S. aureus*” [7]. In addition, the role played by HIV and tuberculosis as causative agents of necrotizing pneumonia in children living in middle- and low-income countries remains to be described. Identification of patients with suppurative parenchymal and pleural complications of pneumonia is important because prolonged intravenous antibiotic treatment is needed and surgical drainage and decortication must be considered [3].

Diagnostic imaging plays a significant role in both the diagnosis and treatment of complications of pneumonia in children [6]. Plain chest radiography represents the imaging modality of choice for several lung diseases, including community-acquired pneumonia [5]. Computed tomography (CT) on the other hand is not a first-line imaging tool for children with suspected uncomplicated community-acquired pneumonia. It is largely reserved for when complications are suspected or where there is difficulty in differentiating community-acquired pneumonia from other pathology. This review will outline the situations where CT needs to be considered in community-acquired pneumonia, describe the imaging features of the parenchymal and pleural complications, discuss how CT may have a larger role to play in developing countries where HIV and tuberculosis are prevalent, note the role of CT scanning when there is a possibility of foreign body aspiration and address radiation concerns.

## CT diagnosis of the suppurative parenchymal complications of pneumonia

**Fig. 1:**
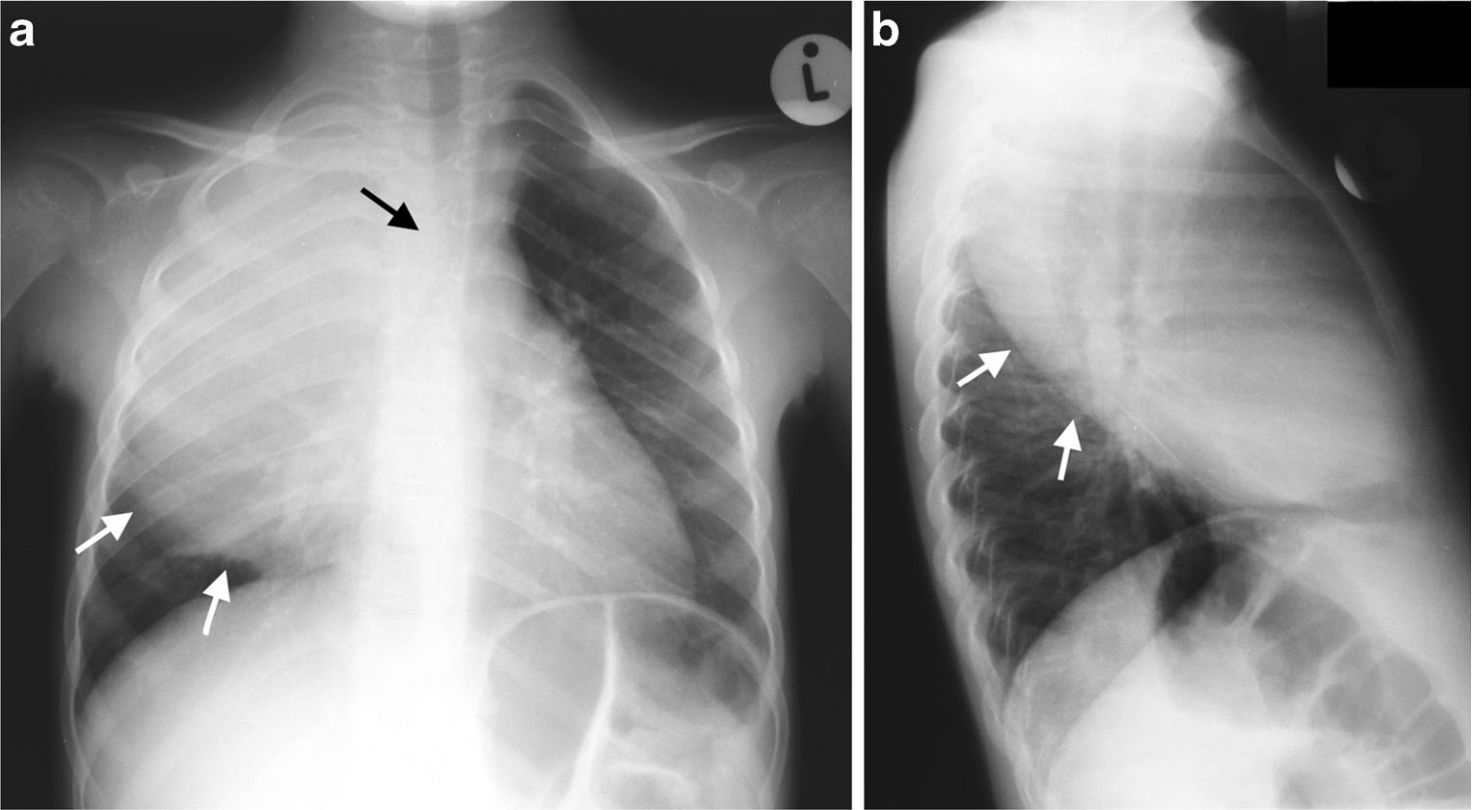
Chest radiographs in a 3-year-old boy who failed to respond to antibiotics and developed expansile pneumonia. Anteroposterior chest radiograph (**a**) and lateral chest radiograph (**b**) demonstrate dense opacity in the right upper lobe, a bulging inferior margin (*white arrows*) and mass effect on the mediastinal structures (*black arrow* in **a**). This was diagnosed as an expansile pneumonia, but the radiograph was unable to distinguish whether the underlying lung was congested or had undergone necrotic or suppurative change. The organism responsible was identified as *Klebsiella pneumoniae*

Expansile pneumonia refers to an increased volume of an involved lobe or segment, with a chest radiograph appearance of dense consolidation causing bulging fissures. Even though expansile pneumonia is not commonly seen in children, *Klebsiella pneumoniae* and *Staphylococcus aureus* are known to cause this, with *Klebsiella pneumoniae* mostly affecting the upper lobes [8] (Fig. 1). *Aspergillus fumigatus* in immunocompromised and neutropenic children [9, 10] as well as *Mycobacteriun tuberculosis* can also cause expansile pneumonia [11]. When there is expansile pneumonia but no visible cavitation containing air or an air-fluid level, the variety of underlying parenchymal changes cannot be differentiated from each other on chest radiographs. These can range from exudative consolidation with fluid bronchograms to necrotizing pneumonia and eventual lung abscess and may be accompanied by a pleural effusion or empyema. These pathological changes can be distinguished on CT, allowing for different management options to be considered. In addition, CT can suggest tuberculosis as a possible cause by demonstrating typical lymphadenopathy or indicate a missed/unsuspected foreign body. Necrotizing pneumonia is a severe complication of community-acquired pneumonia resulting in liquefaction and cavitation of lung tissue [12]. The true incidence of this complication in children with community-acquired pneumonia is unknown, but it is increasingly reported, possibly due to early detection from the increased use of chest CT [7]. Lai et al. [7] reported a frequency as high as 34% using chest CT. Lung necrosis is suspected on chest radiographs, but the diagnosis can only be confirmed by CT [3]. Radiologists are clear that CT yields more detailed information concerning chest anatomy and pathology than chest radiography [2]. In the context of diagnosing necrotizing pneumonia or the other complications of community-acquired pneumonia in children, CT has been shown to be the most sensitive and accurate modality, demonstrating pathology before it becomes evident on chest radiographs [2, 4, 7]. Detection and differentiation of pulmonary parenchymal complications require contrast-enhanced CT [6]. In a study by Donnelly et al. [2], all CT examinations performed in children not responding to treatment for community acquired pneumonia (when a chest radiograph was non-contributory) demonstrated at least one significant finding [2]. The suppurative parenchymal complications of pneumonia seen on CT can be considered an evolution of events from exudation, followed by necrosis of the lung, to cavitation or abscess formation and possibly the development of a bronchopleural fistula, but since CT is not performed in a sequential, temporal fashion in children (a single CT scan only represents a moment in time) and since interventions may interrupt the process, each of these complications can be considered individually.

## CT findings in complicated pneumonia

**Fig. 2:**
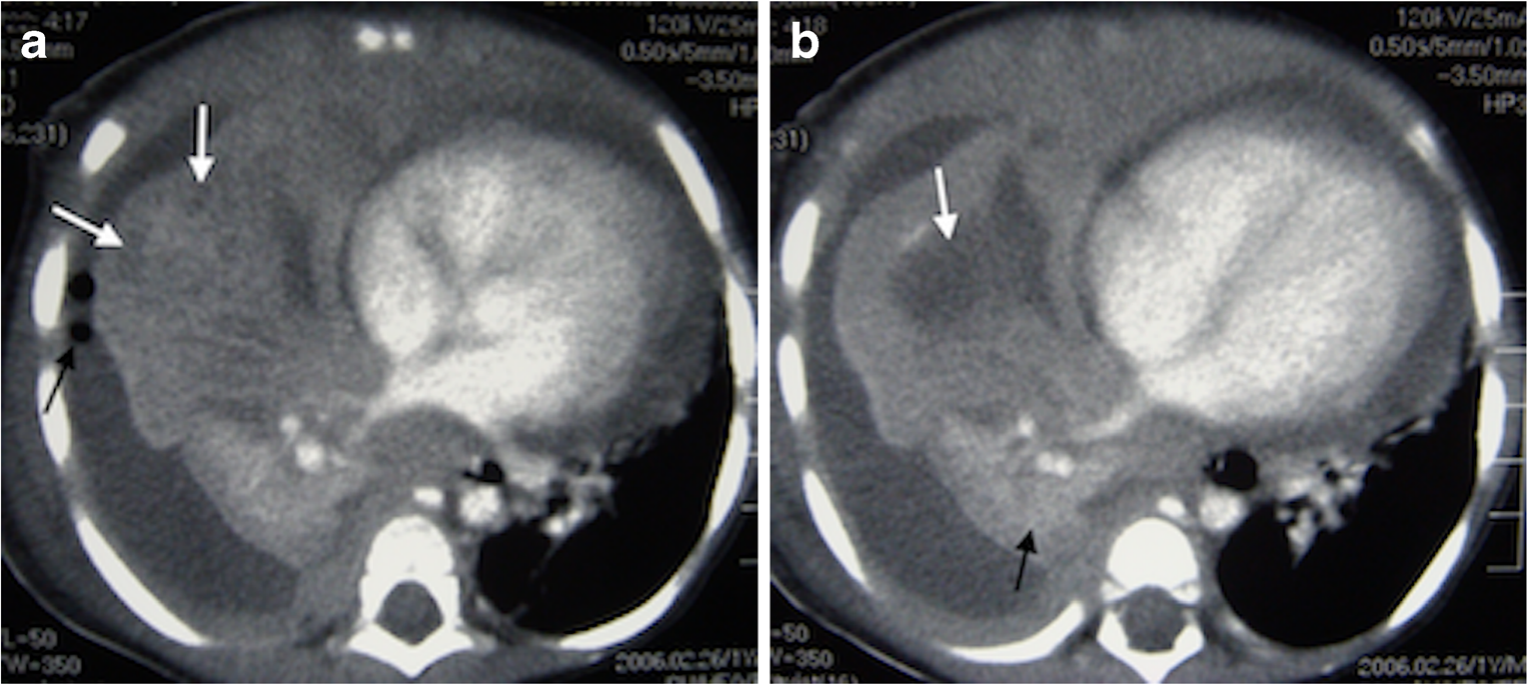
Representative axial contrast-enhanced CT of the chest in a 1-year-old boy with pneumonia, who was not responding to antibiotics. **a** Necrotising pneumonia in the right middle lobe is represented by low density, poorly/non-enhancing or liquefied areas of lung (*white arrows*). In addition, there is a right pleural effusion containing pockets of air *(black arrow)* resulting from an attempted drainage. **b** In contrast to the cavitary process in the centre of the necrotising lung (*white arrow*), the viable consolidated right lower lobe demonstrates enhancement (*black arrow*)

**Fig. 3:**
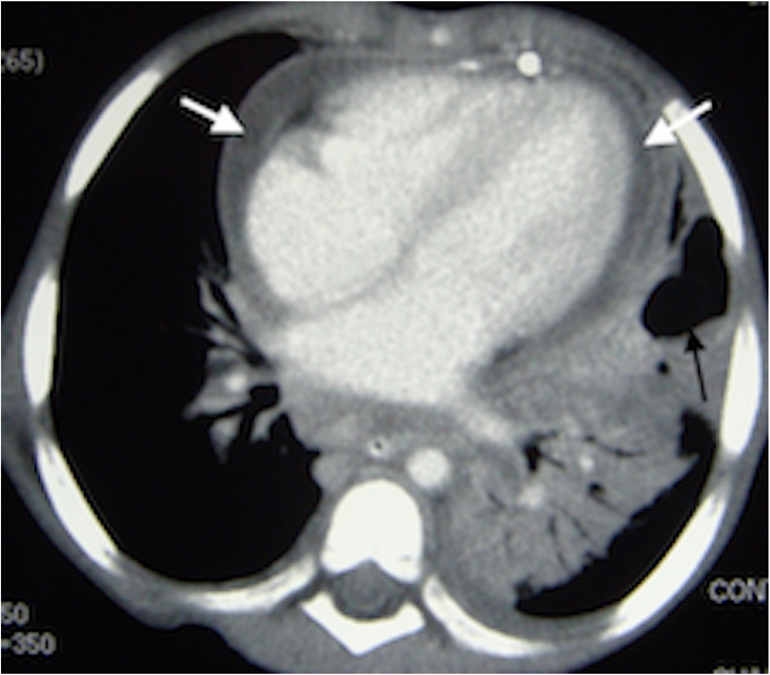
Axial contrast-enhanced CT in a 2-year-old girl with left-side necrotising pneumonia and pericardial collection (*white arrows*) in the process of being drained. There is poor enhancement of the visible portions of the left lung and an air-filled thin-walled cavity without surrounding enhancement representing cavitary necrosis (*black arrow*)

**Fig. 4:**
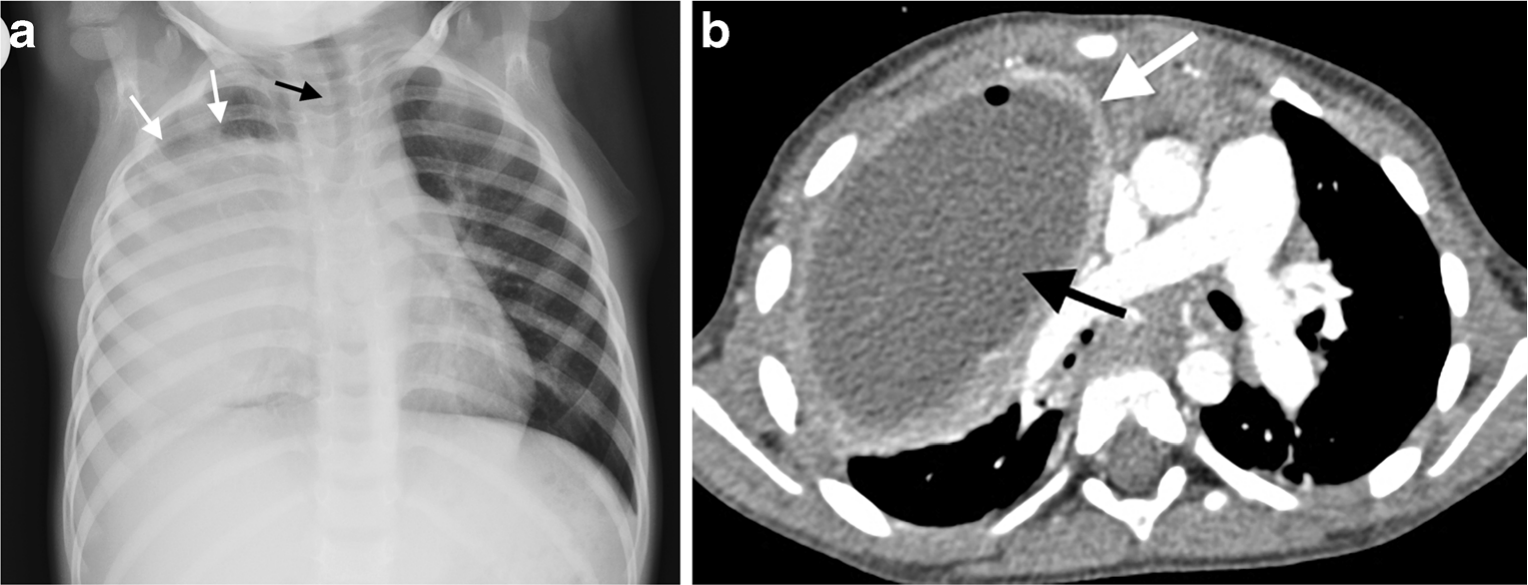
Lung abscess in a 2-year-old boy who failed to respond to antibiotic treatment for pneumonia. **a** Chest radiograph demonstrates an expansile dense opacity in the right lung with outwardly convex superior margin (*white arrows*) and mass effect on the mediastinum (*black arrow*). **b** Axial contrast-enhanced CT demonstrates a large abscess (*black arrow*) in the right lung with a well-defined, thick wall that shows some enhancement (*white arrow*) and displacement of the mediastinum to the left

**Fig. 5:**
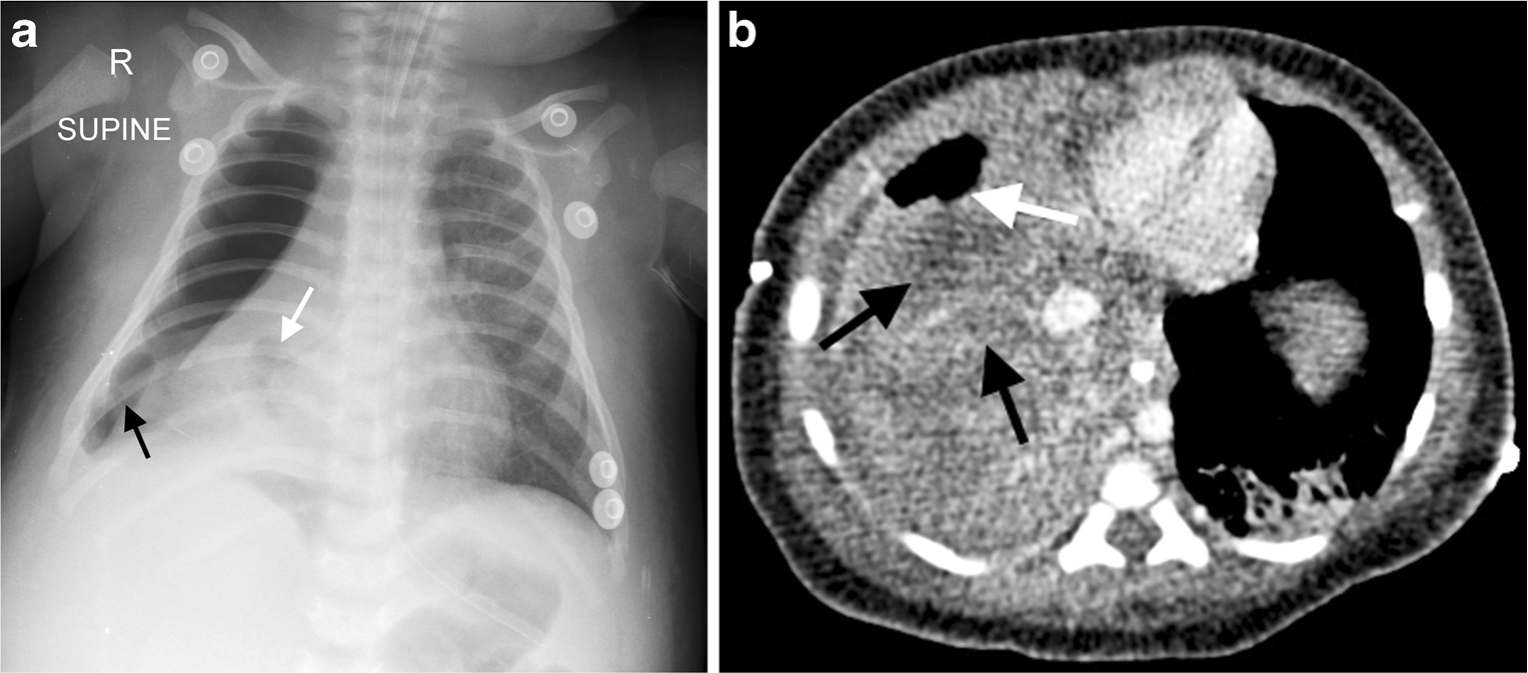
A 3-month-old boy not responding to antibiotics for presumed pneumonia became acutely unwell, short of breath and required intubation **a** The chest radiograph demonstrates dense collapse of the right lung with suspected cavitation (*white arrow*), as well as loculated gas in the pleural space (*black arrow*), suggesting a bronchopleural fistula. **b** Contrast-enhanced axial CT of the chest demonstrates non-enhancing areas of the right lung representing necrotising pneumonia (*black arrows*), and a peripheral cavitated portion of the right middle lobe communicating with the pneumothorax (*white arrow*), in keeping with a bronchopleural fistula

Necrotizing pneumonia is diagnosed on CT when a significant portion of consolidated lung shows diffuse or patchy low attenuation and decreased or no enhancement after intravenous contrast medium administration [2, 6, 7, 13] (Fig. 2). Cavitary necrosis is identified as a dominant area of necrosis with a combination of loss of normal parenchymal lung architecture, decreased parenchymal enhancement and development of multiple thin-walled cavities filled with fluid or air and lacking an enhancing border [2] (Fig. 3). Lung abscess is diagnosed on CT when there is a lung cavity surrounded by a well-defined enhancing wall, no central enhancement and either fluid- or air-filled within [2] (Fig. 4). The distinction between necrotizing pneumonia and lung abscess is based on the visualisation of contrast-enhancing walls of an abscess [4] and is important when entertaining aggressive interventional therapy because this can be therapeutic for lung abscess and harmful for necrotizing pneumonia [14]. A bronchopleural fistula can only definitively diagnosed on CT when a communication between the lung and pleural space is visualized directly [2] (Fig. 5). Donnelly et al. [2] reported that of the 56 CT scans performed in children with community-acquired pneumonia failing to respond to treatment, there were 40 with parenchymal complications (28 with decreased parenchymal enhancement or cavitary necrosis*,* 5 with abscess and 5 with bronchopleural fistula), 37 with pleural complications and 13 with pericardial effusion.

## Pleural complications of pneumonia

Pneumonia can also be complicated by pleural effusions that do not resolve with antibiotic therapy and may require surgical drainage or thoracoscopy [2, 3]. It is estimated that while 1% of children with community-acquired pneumonia develop pleural effusions, effusions are seen much more frequently in hospitalized children (up to 40%). According to the British Thoracic Society guidelines [3], when there is persisting fever despite adequate antibiotic treatment, clinicians should suspect the development of an empyema. Lai et al. [7] also report an incidence of 12% to 66% of bronchopleural fistula developing as a complication of necrotizing pneumonia.

**Fig. 6:**
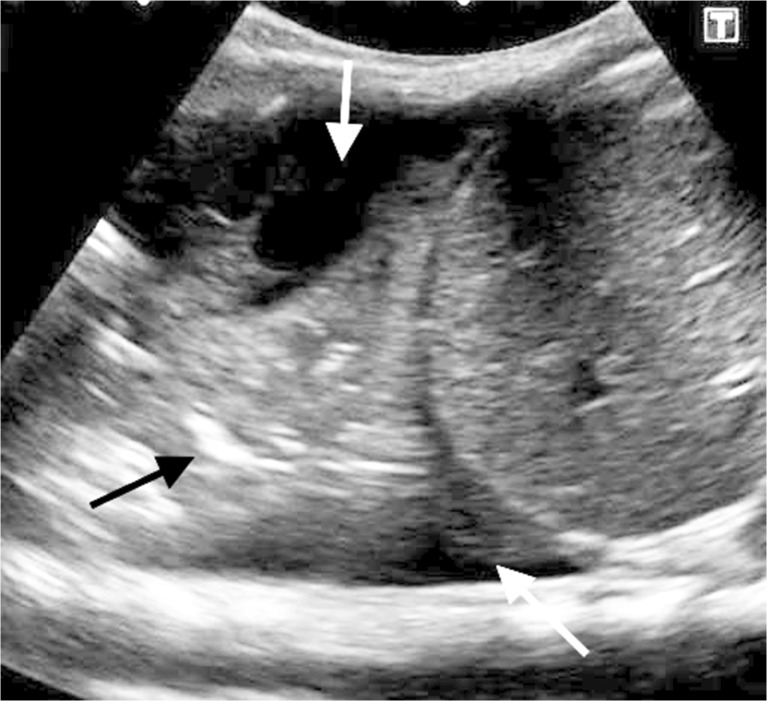
Longitudinal US of the right chest in a 6-year-old girl demonstrates a large, uncomplicated effusion (*white arrows*) surrounding a consolidated underlying lung (*black arrow*) and filling the costophrenic angles, anteriorly and posteriorly. The patient was referred for drainage because of the size of the effusion and the symptoms

**Fig. 7:**
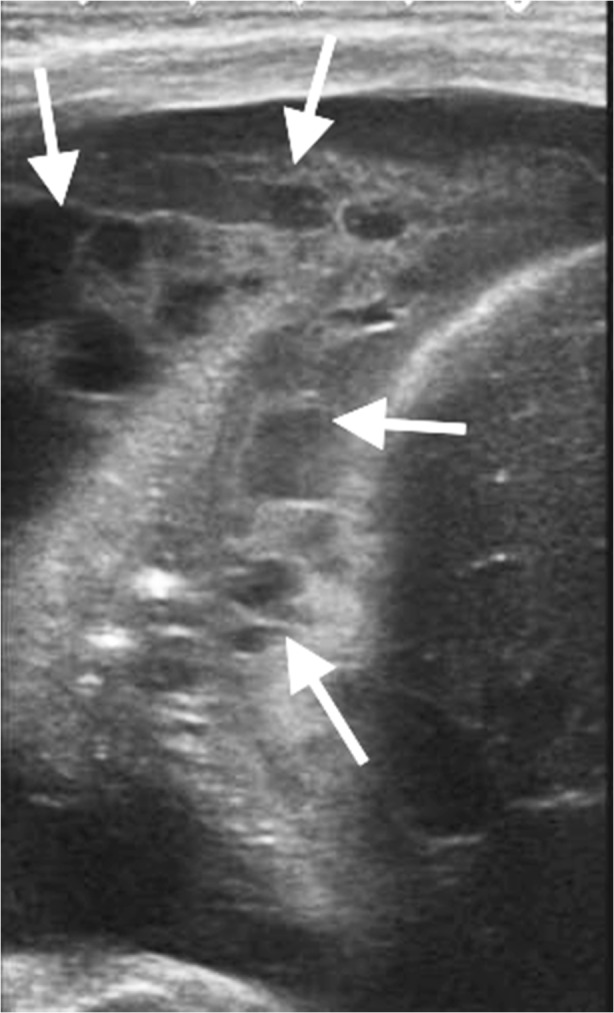
Longitudinal US of the right chest in a 5-year-old boy confirms a clinically suspected empyema by demonstrating a complex effusion containing internal loculations and fibrin strands (*white arrows*). There was no movement of the lung edge and the patient required surgical intervention

**Fig. 8:**
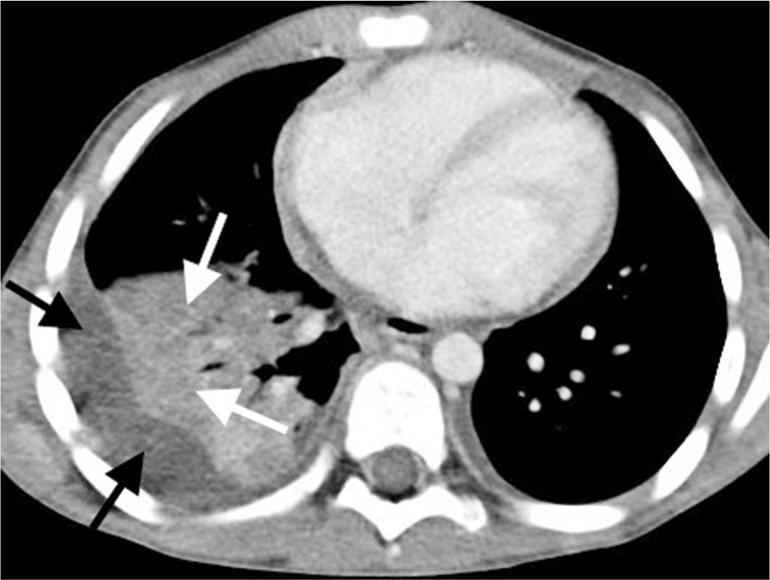
Contrast-enhanced axial CT of the chest in a 5-year-old boy demonstrates an effusion (*black arrows*) associated with an air-space process. The patchy low-density areas with less enhancement in the consolidated lung are in keeping with necrotising pneumonia (*white arrows*) and the patient required prolonged antibiotic treatment

**Fig. 9:**
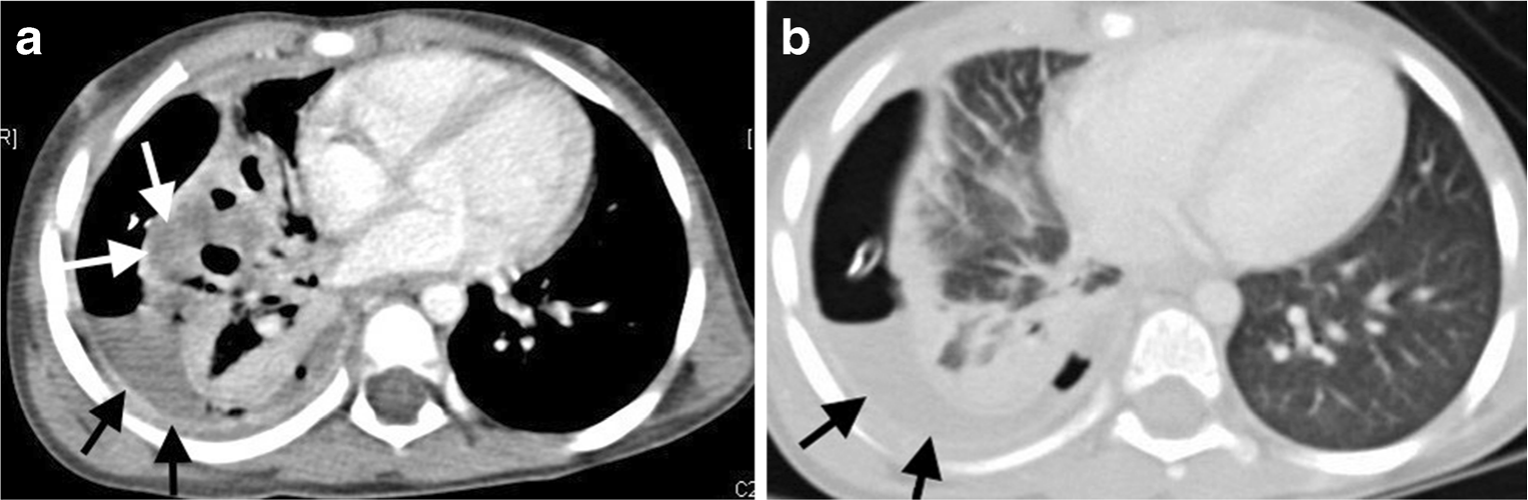
A 6-year old girl with empyema, who has an indwelling right-side chest drain. Contrast-enhanced axial CT of the chest in soft-tissue window (**a**) and lung window (**b**) demonstrate that the lung underlying the empyema (*black arrows*) has patchy areas of non-enhancement/low density (*white arrows* in **a**) indicating necrotising pneumonia without cavitation

**Fig. 10:**
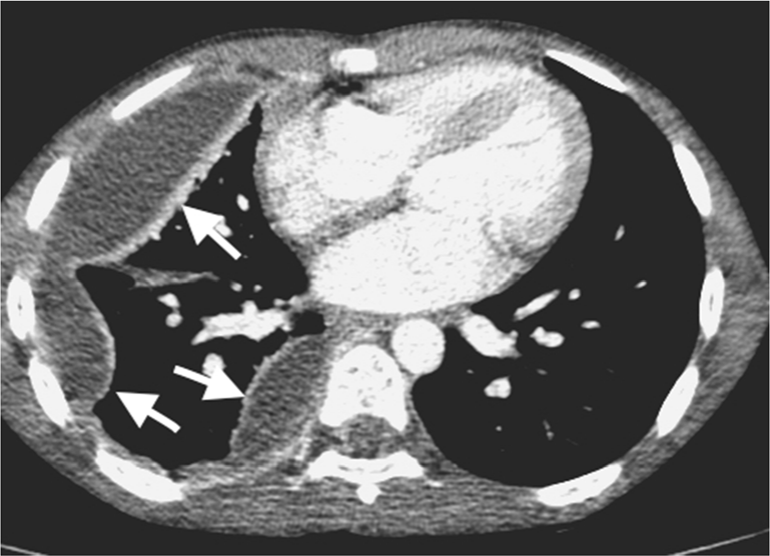
Contrast-enhanced axial CT of the chest in a 9-year-old girl demonstrates a thick enhancing rim associated with loculated pleural collections (*white arrows*), indicating an empyema, but there is no underlying parenchymal abnormality

Even though pleural effusion is visible on chest radiographs, ultrasound (US) is the recommended method for estimating the amount of fluid [3] (Fig. 6). The size of the effusion is significant when deciding on management [5]. Chest US is also considered superior to chest CT in its ability to demonstrate internal components of an effusion such as loculations and fibrin strands [6, 15] (Fig. 7). The British Thoracic Society guidelines recommend chest US for detecting pleural effusion and guiding drain placement, but indicate that CT with intravenous contrast ‘‘is useful for evaluation of advanced parenchymal disease’’ associated with effusions [6, 16] (Figs. 8 and 9). The Pediatric Infectious Diseases Society and the Infectious Diseases Society of America note that even though history and physical examination can suggest the possibility of an accompanying effusion in children with community-acquired pneumonia, chest radiographs should be used for confirmation while chest US or CT should only be performed when chest radiography is inconclusive [5] (Fig. 10). Identifying any parenchymal complication such as necrotizing pneumonia or abscess coexisting with an effusion is important because these findings will ensure treatment with a prolonged course of antibiotics [6, 17].

Chest CT is also often performed to delineate the anatomy [6, 16] and for planning all types of surgical treatment including “chest tube drainage (with or without thrombolytics), video-assisted thoracoscopy, or open thoracotomy and decortication” [6].

Bronchopleural fistula develops as a complication of pneumonia if lung necrosis extends through the pleura. Bronchopleural fistula is associated with higher morbidity [18] (Fig. 5).

## HIV-infected children

**Fig. 11:**
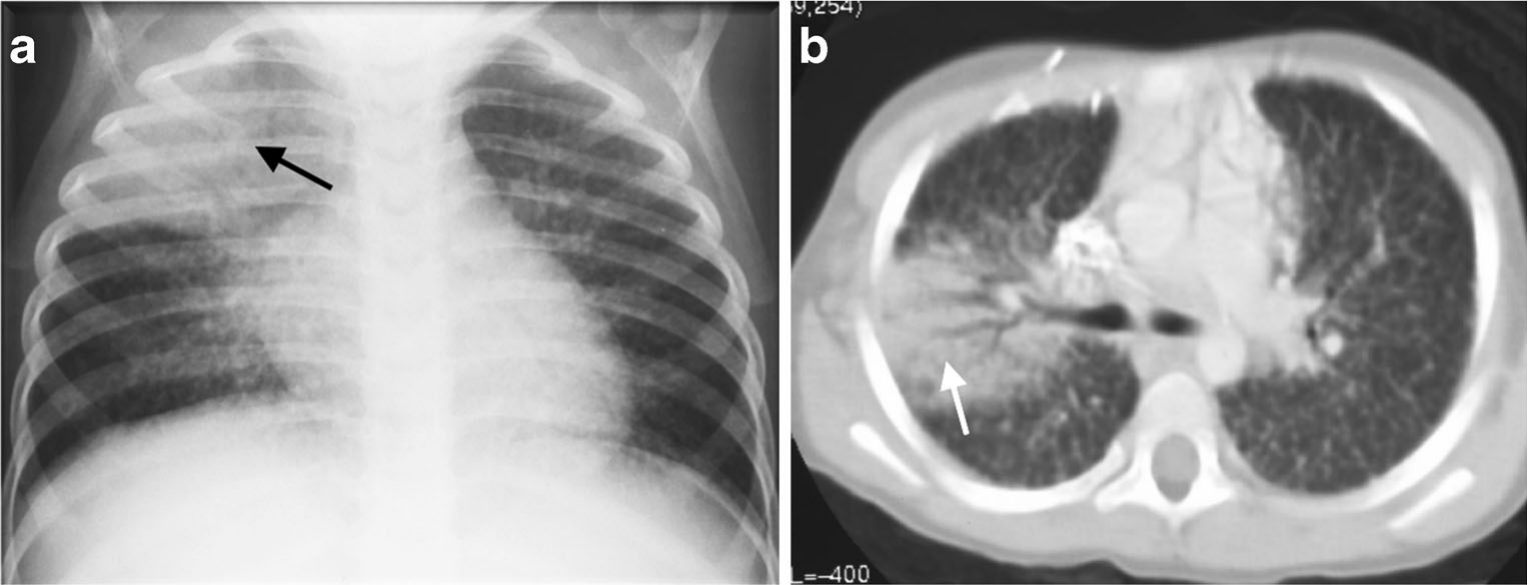
A 5-year-old boy with HIV and an acute episode of pneumonia. **a** Anteroposterior chest radiograph demonstrates a right upper lobe area of air-space consolidation (*arrow*) and also demonstrates widespread small nodules in both lungs. **b** Contrast-enhanced axial CT confirms both the air-space process in the right lung (*arrow*) and the widespread nodules affecting the interstitial component of the lung parenchyma and resulting in a fine lacework pattern, typical of HIV-associated lymphoid interstitial pneumonitis

Chest CT scanning is useful in HIV-infected children with acute pneumonia, as many of them have underlying chronic lung disease, including bronchiectasis, or because they may be infected with more than one organism. Many of these children live in areas with a very high incidence of tuberculosis and may either have tuberculosis or a combination of an acute pneumonia and tuberculosis. The symptoms of tuberculosis in HIV-infected children may overlap with signs and symptoms of other infections as well as HIV itself and tuberculosis may therefore be confused with acute pneumonia [19–21]. Older HIV-infected children may have also developed lymphoid interstitial pneumonitis, which makes them more vulnerable to acute chest infections [22]. Chest CT scan, therefore. is indicated for diagnosing lymphoid interstitial pneumonitis and features of tuberculosis if it is considered in the differential diagnosis (Fig. 11). Lymphadenopathy associated with lymphoid interstitial pneumonitis does not have the classic rim or ring–like enhancement seen in tuberculosis [23, 24]. The importance of polymicrobial infections in acute pneumonia is well recognized and a significant number of pathogens have been reported as causes of pneumonia in HIV-infected children [25], including combinations of bacterial, viral, *Pneumocystis jirovicii* and mycobacterial infections. The most common causes of acute bacterial pneumonia in the HIV-infected group remain *Streptococcus pneumonia* and *Staphylococcus aureus*; amongst gram-negatives, the *Klebsiella* species, *Haemophilus influenza*, *Escherichia coli* and *Salmonella Pseudomonas* species are important and Mycoplasma is also found [25]. Mortality is increased significantly with increasing numbers of pathogens -- children with polymicrobial pneumonia have a 10-fold greater risk of dying [26]. Because HIV-infected children with pneumonia are more likely to have severe disease and because the rate of complications is higher in HIV-infected children [26], the threshold for performing CT scanning (where available) should be lower than that recommended for children with community-acquired pneumonia who are not immunocompromised.

## Tuberculosis - parenchymal complications

**Fig. 12:**
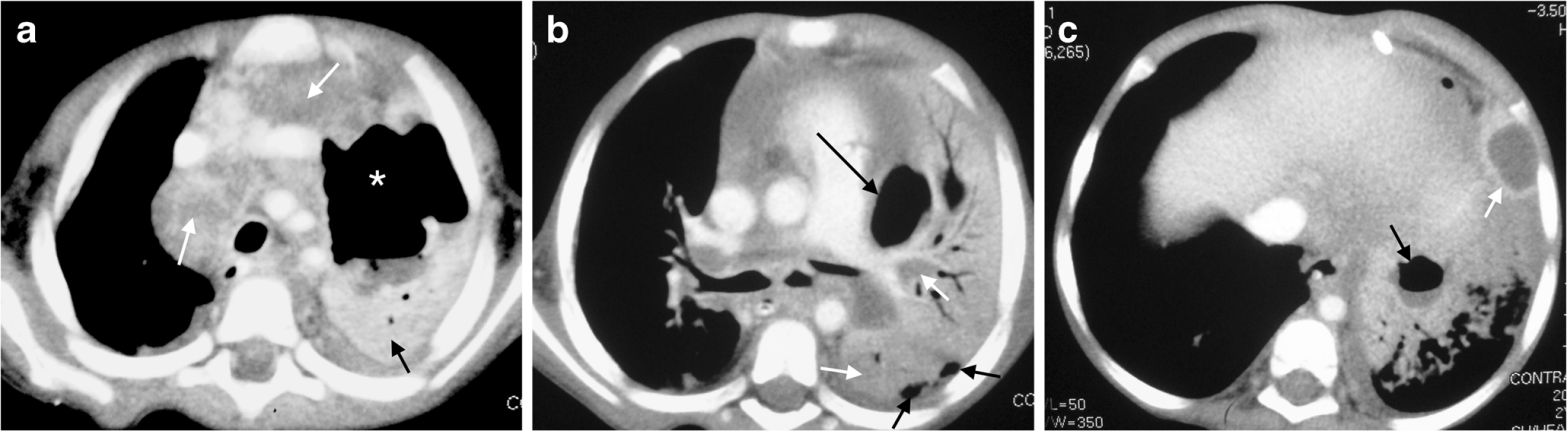
A 2-year-old girl with confirmed tuberculosis, demonstrating progression of lung necrosis. **a** Axial CT at the level of the main branches of the aorta demonstrates the typical low-density right paratracheal and anterior mediastinal lymphadenopathy (*white arrows*), associated with tuberculosis as well as a large air-filled cavity (*asterisk*) and fluid level in the left upper lobe due to lung necrosis. Some consolidated vital lung is seen enhancing posteriorly (*black arrow*). There is also bilateral effusion. **b** Axial CT at the level of the main pulmonary outflow tract demonstrates medial and posterior low density non-enhancing necrotic areas of the left consolidated lung (*white arrows*) as well as a medial cavity very closely associated with the pleural space (*long black arrow*). There are also small pockets of air in the pleural space posterior (*short black arrows*). **c** Axial CT at the level anterior diaphragm demonstrates cavitation of the left lower lobe with an air-fluid level (*black arrow*) as well as formation of an abscess seen as a clearly enhancing wall, surrounding a fluid collection (*white arrow*)

Expansile pneumonia caused by *M. tuberculosis* is difficult to distinguish from other causes of expansile pneumonia because the characteristic tuberculous hilar lymphadenopathy is often masked by the parenchymal disease on chest radiographs. The upper lobes are involved in 75% of such cases. Chest CT may be diagnostic of tuberculosis when there is one of three possible patterns: (1) dense homogeneous opacification with no evidence of liquefaction of the affected lobe and patent airways seen as air bronchograms, (2) homogeneous opacification with areas of necrotic liquefaction with visible glandular obstruction of the airways and absence of air bronchograms or (3) a combination of (1) and (2) [11] (Fig. 12).

Children with tuberculosis may rarely present with mediastinal abscess. These lesions cannot be diagnosed on chest radiographs, but CT scan may demonstrate a mass of peripherally enhancing lymphadenopathy that has undergone central breakdown and developed the appearances of an abscess [27]. These children may present with swinging fever, may not respond to routine antibiotics, and even on tuberculosis treatment may take a very long time to respond.

## Foreign body aspiration

**Fig. 13:**
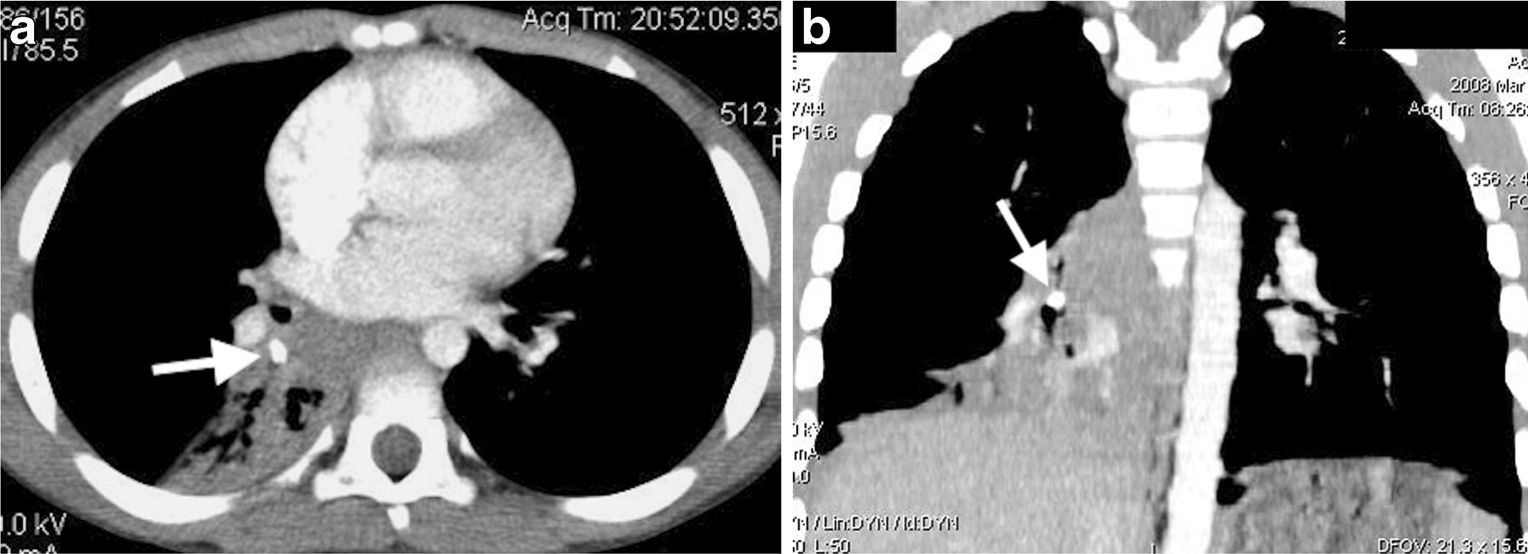
A 9-year old boy with a 6-month history of cough. Contrast-enhanced CT performed for a persistent right lower lobe air-space opacification on chest radiographs demonstrates a dense foreign body (*white arrows*) on axial section (**a**) and coronal reconstruction (**b**) as a cause of the right lower lobe posterior segment atelectasis

Foreign body aspiration often presents with nonspecific respiratory signs and symptoms, which result in diagnostic delays and lead to chronic respiratory morbidity [28]. Even though it is not as common as in adults, bronchial obstruction (e.g., from an aspirated foreign body) should be considered as a cause of persistent pneumonia in children [2]. The diagnosis should also be considered in patients with acute pneumonia, especially if there is significantly reduced ventilation in the area of involvement as seen on chest radiography. Definitive diagnosis of foreign body aspiration is with bronchoscopy, but in the developing world the lack of access to bronchoscopy services [29] means that CT scan may be the main diagnostic modality, especially since organic foreign bodies will not be visible on the plain chest radiographs (Fig. 13). Rarely, children with foreign body aspiration may present with empyema and bronchopleural fistula. In these cases, CT scan is necessary to determine the type and time frame of the intervention that will be needed [30].

## Radiation concerns and CT

CT enables detailed analysis of lung anatomy in a similar manner to gross pathology sections but is burdened by a higher radiation dose as compared to chest radiographs. Therefore, the diagnostic benefit and impact on patient management must be weighed against any possible risks. Due to the high diagnostic information that is provided, CT has been utilized in an enthusiastic manner within the last decades – causing it to be a major source of collective radiation dose [31, 32]. This CT overuse instigated a closer look at the doses imparted [33]. Because of the higher radiation sensitivity reported for children as compared to adults, radiation-related cancer has become of particular concern [34]. Therefore, currently there is a tendency to withhold CT from children despite its high diagnostic impact and problem-solving capability. Additionally, there are only sparse recommendations/indications listed for CT, especially in the context of childhood pneumonia [35]. Use of CT in a reasonable manner and through adjustment of scan parameters for pediatric needs allows CT to be applied safely in children. In the following paragraphs, the most important factors influencing CT dose will be listed and discussed as well as simple ways to reduce the radiation burden. A broader overview of the topic is provided in the publication by Sorantin et al. [31]. Furthermore, a rough dose comparison between chest radiographs and CT is provided.

There are several factors influencing CT dose. Appropriate slice thickness selection is a simple way to decrease the dose. There is an inverse relationship between slice thickness and dose for the same image quality. This means that for a given image quality, doubling the slice thickness will result in 50% of the dose. In almost all age ranges, 5-mm slice thickness represents a good choice. Appropriate selection of tube voltage setting represents another challenge. Modern CT systems offer a voltage range from 70 kV to 140 kV. Dose and kV are connected by a quadratic dependency, thus reducing tube voltage from 120 kV to 80 kV in small children, offering a dose reduction of more than 50%. Additionally, due to the changed attenuation (less hard radiation), the inherent tissue contrast is enhanced. A good starting point is 70–80 kV in children up to 2 years old, progressing to 100 kV until adolescence and then increasing to 120 kV in adulthood. Today due to “automatic exposure control” techniques, tube current is fixed by preselecting an explicit image quality – e.g., in some systems this is achieved by reference mA and in others by a noise value. Furthermore, iterative image reconstruction techniques offer another possibility to save approximately 50% of dose as compared to filtered back projection [36–38]. The chest is a high-contrast region (air-filled lungs, mediastinal soft tissue, bones) as compared to the abdomen. Therefore, as a rule of thumb, the dose in chest CT should be only 66% of abdominal CT or abdominal CT should have 50% more dose than a chest CT.

The radiation dose of CT has been summarised in several studies including a recent one from Italy by Granata et al. [39]. Applying conversion factors, as released by the ICRP paper 103 [40], to the dose length product (DLP), values from the study by Granata et al. [39] allow the effective dose to be approximated. These data are presented in Table 1 alongside data from a 2016 survey at the institution of one of the authors (E.S.). Since some information from the Italian paper was not available, the effective dose was calculated using the conversion factors for three kV settings (80–120 kV). Chest radiography is one of the most frequently performed imaging studies and therefore a dose comparison between chest radiographs and CT is appropriate. A reasonable, effective dose estimation for an anteroposterior (AP) chest radiograph is 0.01 mSv [41]. According to Table 1, chest CT can be performed in children up to adolescence with an effective dose of 1.0 mSv and therefore corresponds to about 100 AP chest radiographs. The dose for a lateral is approximately two times the dose of an anteroposterior chest radiograph, which would mean that a combination AP and lateral would be 0.03 mSv. The full potential for dose savings in CT is not yet determined and some researchers are experimenting with chest CT examinations at the dose of radiography [42].

**Table 1:** Approximate effective dose for chest CT. Dose values published [39] as well as data from a recent study at one author’s (E.S) institution

		Effective dose (mSv) in chest CT	
Age	kV	Data from Granata et al. [39]	Data from the Department of Radiology, Medical University Graz, Austria
< 6 yrs	80	1.68	0.77
	100	1.54	0.71
	120	1.49	N/A
6–10 yrs	80	2.30	N/A
	100	2.16	1.07
	120	2.10	N/A
≥11–15 yrs	80	3.10	N/A
	100	2.94	2.51
	120	2.93	2.50

*N/A* not available – these tube voltage settings are not used at that institution. A chest CT can be performed in children up to adolescence with an effective dose up to 1.0 mSv

Questions arise as to how the optimal dose can be determined. A very simple approach termed the *half slice thickness approach* has been devised and is in use by the Department of Radiology, Medical University Graz, Austria. This approach does not need any investment in phantoms or measuring devices. Starting with the standard settings, patients are scanned and images reconstructed in the usual way. Subsequent to this, images should be reconstructed with only half of the chosen slice thickness. Due to the above-mentioned relationship between noise and slice thickness, these images will appear noisier. If the radiologist still finds these images diagnostic, then the excess dose is 100%. At the next examination, the dose is lowered by 20% (e.g., reference mA settings) and the procedure is repeated. After a few examinations, the radiologist will refuse to lower the dose and it is at this point that the optimal dose for that CT protocol is established. A further possibility to save radiation is to target the examination to the clinical question. At follow-up examinations of complicated community-acquired pneumonia (e.g., for abscess, empyema), the tube current can be further reduced (e.g., 30–50% off), since the underlying process is already known and it is possible that the follow-up CT scan is only required to document resolution or any residual disease. New CT technology allows for faster scans at much lower doses, thereby also avoiding the need for anaesthesia and allowing for follow-up scanning to be performed safely, in selected cases [13].

## Conclusion

CT needs to be performed at acceptable radiation doses in children and is indicated for diagnosing complications associated with pneumonia, when patients fail to respond to treatment, when chest radiographs are suggestive, when empyema is diagnosed on US and in environments where HIV and tuberculosis are likely coinfections. Even though US is adequate for demonstrating and evaluating effusions, CT is important for demonstrating the underlying lung in search of associated necrosis or abscess formation. CT is superior to chest radiographs and the relatively low theoretical cancer risks need to be appropriately considered against the many advantages it affords the clinician, so as not to deny children the benefits of rapid and accurate diagnosis.
